# Hyperphosphorylation of Intrinsically Disordered Tau Protein Induces an Amyloidogenic Shift in Its Conformational Ensemble

**DOI:** 10.1371/journal.pone.0120416

**Published:** 2015-03-13

**Authors:** Shaolong Zhu, Agnesa Shala, Alexandr Bezginov, Adnan Sljoka, Gerald Audette, Derek J. Wilson

**Affiliations:** 1 Chemistry Department, York University, Toronto, ON, Canada; 2 Department of Medical Biophysics, University of Toronto, Toronto, Canada; 3 Department of Physics, Ryerson University, Toronto, ON, Canada; 4 Center for Research in Mass Spectrometry, Faculty of Science, York University, Toronto, ON, Canada; University of South Florida College of Medicine, UNITED STATES

## Abstract

Tau is an intrinsically disordered protein (IDP) whose primary physiological role is to stabilize microtubules in neuronal axons at all stages of development. In Alzheimer's and other tauopathies, tau forms intracellular insoluble amyloid aggregates known as neurofibrillary tangles, a process that appears in many cases to be preceded by hyperphosphorylation of tau monomers. Understanding the shift in conformational bias induced by hyperphosphorylation is key to elucidating the structural factors that drive tau pathology, however, as an IDP, tau is not amenable to conventional structural characterization. In this work, we employ a straightforward technique based on Time-Resolved ElectroSpray Ionization Mass Spectrometry (TRESI-MS) and Hydrogen/Deuterium Exchange (HDX) to provide a detailed picture of residual structure in tau, and the shifts in conformational bias induced by hyperphosphorylation. By comparing the native and hyperphosphorylated ensembles, we are able to define specific conformational biases that can easily be rationalized as enhancing amyloidogenic propensity. Representative structures for the native and hyperphosphorylated tau ensembles were generated by refinement of a broad sample of conformations generated by low-computational complexity modeling, based on agreement with the TRESI-HDX profiles.

## Introduction

Full length tau protein (htau40) and it’s splice variant isoforms [1] were originally identified in their normal biological role as promoters of cytoskeletal stability through specific interactions in microtubules [2]. Great interest in tau was briefly ignited when it was found to be one of the two main amyloidogenic species in Alzheimer’s [3], the other being the Amyloid-β (Aβ) peptide. However, interest waned somewhat when it was discovered that familial Alzheimer’s was linked to mutations exclusively affecting Aβ. In recent years, tau has again become the focus of intensive interest, partly due to a growing sense that its role in Alzheimer’s may have been underappreciated [4], but also as a consequence of its central role in a host of other neurodegenerative disorders, including a set of conditions known collectively as tauopathies [5]. The mechanisms driving tau pathology are poorly understood, with often conflicting evidence for splice variant ratios [1,6], total concentration [7], non-physiological interactions with vesicles [8,9], misprocessing [10] and erroneous post-translational modification [11]. In recent years, the role of mis-phosphorylation, particularly hyperphosphorylation by GSK-3β has emerged as a significant occurrence in tau pathology [12–14]. However, without an understanding of the structural implications of hyperphosphorylation, it is unclear if it is a causative agent [11,12,15], a cellular response to amyloid formation [14], or even a protective mechanism against pre-fibrillar aggregate toxicity [16].

Intrinsically disordered proteins and domains have long represented a challenge to structural biologists [17]. Disordered proteins often fail to crystalize and typically do not provide sufficient electron density to calculate an X-ray crystallographic structure when they do. NMR can provide significant insights in some cases, particularly with respect to transient secondary structure (*via* chemical shift index analysis) [18] and occasionally weak tertiary contacts [19]. However, the lack of a well-defined native structure tends to broaden-out signals or cause low dispersion in NMR spectra, making conventional structural NMR analyses exceedingly challenging (and often impossible) [20]. Nonetheless, intrinsically disordered proteins do exhibit ‘residual structure’, corresponding to biases in their native conformational ensembles that provide a basis for specific functional properties. The potential to conduct structure/function analyses on IDPs provides strong motivation to characterize residual structure, however, there is presently no widely-used analytical approach to achieve this, especially in the case of large proteins (> 200 residues) which generally cannot be analyzed by NMR [18].

Hydrogen/Deuterium eXchange (HDX) is a structure-dependent labeling technique that is well-suited to probing conformational dynamics and activity-linked structural changes in proteins including folding [21], ligand/binding [22] and catalysis [23]. The underlying principle of this approach is that hydrogens on protein amide, hydroxyl or thiol groups undergo exchange with hydrogens from solvent (usually water). If the solvent hydrogens are replaced by deuterium, exchange results in deuterium uptake on the protein at site-specific rates that are determined by (**i**) the adjacent amino acids (primary sequence), (**ii**) solvent access and (**iii**) hydrogen bonding. For an excellent, thorough review of protein HDX, the reader is directed to ref. [24]. The principle advantage of HDX is that even structures that are populated very briefly or rarely are reflected in the deuterium uptake profile, allowing for the characterization of transient, weakly-populated conformers in native structural ensembles. However, with a very small number of exceptions [25,26], HDX has not been used to study IDPs because weak hydrogen bonding and rapidly fluctuating tertiary structure results in complete (or near-complete) exchange prior to the first measurement.

**Fig 1:**
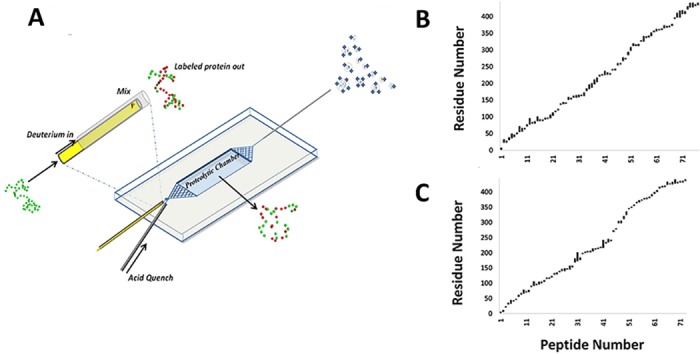
TRESI-HDX chip and digestion profiles. **A**. Schematic depiction of the device used to carry our time-resolved HDX measurements using a ‘bottom-up’ workflow. The protein sample is mixed with D_2_O in a variable-position capillary micromixer (inset), followed sequentially by a delay corresponding to 42–8000 ms of exchange (depending on the position of the mixer), acid-quenching of the HDX reaction, pepsin-based digestion and on-chip ESI for transfer of labeled peptides into the gas phase. **B** and **C** Digestion profiles for native tau (**B**) and hyperphosphorylated tau (**C**). The profiles are roughly linear in shape, suggesting that there are no regions of tau that are particularly resistant to proteolysis by pepsin (resistant regions, which can arise from the presence of secondary or tertiary structure at low pH, would appear as verticle ‘jumps’ in the profile).

In this work, we employ a straightforward and broadly-applicable technique based on Time-Resolved ElectroSpray Ionization (TRESI) Mass Spectrometry and HDX that provides a detailed picture of residual structure in IDPs and the shifts in conformational bias that mediate their function. In contrast to conventional HDX experiments, which reflect mainly the stability of hydrogen bonding in structured regions [27,28], TRESI-HDX is sensitive to *weak* hydrogen bond networks and solvent accessibility [26], both of which are modulated by residual structure in IDPs. Analysis of the data is exceedingly straightforward: Regions of the protein where backbone amide protons are transiently hydrogen bonded or sequestered from solvent (*i*.*e*., where residual structure is present) will exhibit an attenuated rate of deuterium uptake compared to the known ‘random coil’ rates on the millisecond time-scale [29]. For site-specific measurements, this is often expressed quantitatively as protection factor PF, which is a ratio of the random coil rate *k*_rc_ to the observed rate *k*_obs_, *i*.*e*., PF = *k*_rc_/*k*_obs_ (S1 Fig.). In the ‘bottom-up’ HDX workflow used in the present study, the individual PFs are averaged over short protein segments [28]. The data therefore yield a semi-quantitative profile indicating the location and relative extent of residual structure, with an average spatial resolution that depends on how efficiently the labeled protein is digested prior to ionization (in our setup, digestion occurs in a microfluidic reactor, see Fig. 1A).

To understand the structural basis of tau pathology, we have characterized the native ensemble of full-length tau, providing a detailed account of the conformational bias shifts that occur upon hyperphosphorylation by the kinase GSK-3β. The structural changes observed are easily rationalized as increasing amyloidogenic propensity, suggesting that GSK-3β mediated hyperphosphorylation is a causative event in tau amyloidogenesis.

## Materials and Methods

### Expression and purification of htau40

Tau expression was carried out as previously reported [30]. Briefly, pET-29b tau plasmids containing htau40 isoform [2] were transfected into *E*.*coli* BL21 cells for protein expression. Bacteria were grown at 37°C to an optical density of 0.5 at 600nm absorbance. The bacterial cultures were then induced using isopropyl beta-d-thiogalactopyranoside (IPTG) for additional 3.5 hours. Cells were pelleted by centrifugation at 6000xg for 30 minutes at 4°C. The pelleted cells were resuspended in resuspension buffer: 20mM MES, 0.2mM MgCl2, 5mM DTT, 1mM PMSF and 1x of halt protease inhibitor cocktail (1mM AEBSF• HCl, 80nM Aprotinin, 5μM Bestatin, 1.5 μM E-64, 2μM Leupeptin, 1 μM Pepstatin A), pH 6.8. Lysates were prepared by sonication on ice for total of 20 minutes (15s on/30sec off). The sonicated lysate was then incubated in boiling water for 20 minutes to denature most of the proteins apart from tau. Cell debris and denatured proteins were pelleted by centrifugation at 40,000xg for an hour at 4°C. The supernatant was dialysed three times for a day against 1L of cation exchange loading buffer (Buffer A): 20mM MES,50mM NaC1,1 mM MgC12, 2mM DTT, 1mM PMSF, pH 6.8. Tau was then purified using cation exchange column: SP Sepharose FF using akta purifier system. Unbound proteins were washed out with 5 column volumns (CV) of buffer A. Tau protein was eluted using cation exchange elution buffer (Buffer B): 20mM MES,1M NaC1,1 mM MgC12, 2mM DTT, 1mM PMSF, pH 6.8 with step gradient of 10% in 2 CV, 15% in 2 CV, 20% in 3 CV, 25% in 3 CV, 30% in 3 CV, 35% in 3 CV, 40% in 3 CV and 100% in 6 CV. The purified tau from cation exchange is pooled and concentrated by vivaspin; 10kDa MWCO to final volumn of 1.5 to 3 ml. The protein concentration is determined by BCA assay. The stock protein was then aliquoted and stored in -80°C.

### Expression and purification of GSK3β

Expression of GSK3β was carried out as previously reported with a few minor modifications [31]. GSK3β-pGEX plasmids (generously supplied by Dr. Jim Woodgett) were transfected into *E*.*coli* BL21 cells for protein expression. Bacteria were grown at 37°C to an optical density of 0.5 at 600nm absorbance. The bacterial cultures were then induced using isopropyl beta-d-thiogalactopyranoside (IPTG) for additional 4 hours. The pelleted cells were resuspended in wash buffer: 30mM Tris-HCl, 100mM NaCl, 0.1mM EGTA, 0.1mM EDTA, 0.25mM DTT, pH 7.3. Lysates were prepared by sonication on ice for total of 10 minutes (15s on/30sec off). Lysates were then pelleted by centrifugation at 40,000xg for an hour at 4°C. The supernatant was then loaded onto the GST-gravity column and the desired product was eluted using wash buffer with 10mM glutathione. The protein was then concentrated and buffer exchanged with wash buffer using 10kDa MWCO vivaspin tubes. The stock proteins were then aliquoted with addition of 20% glycerol and was stored in -80°C.

### Tau phosphorylation

100μM of tau was incubated with 2.5–5 μM (5000 units) of GSK3μ in phosphorylation buffer containing 50mM Tris-HCl, 10mM MgCl_2,_ 5mM DTT and 3mM ATP, pH 7.5 in 30°C. Tau was maximally phosphorylated by GSK3μ in 30 hours [32,33].

### TEM analysis of fibrillar aggregates

Unphosphorylated and hyperphosphorylated tau were further incubated at room temperature to allow formation of fibrillar aggregates, which were observed after 8 days and 48hrs, respectively. TEM images were acquired using a Philips EM201 Accelerating Electron Microscope operated at 80 kV. 5μl of protein solutions were blotted on 100 mesh copper grid with formvar coating for 10 minutes. The samples were then stained with 2.0% uranyl acetate for 5 minutes. The samples were observed under magnification of 30k-100k.

### Protein Identification using MS

Aliquots of concentrated native tau was buffer exchanged with 5kDa MWCO dialysis cassette in 50mM ammonium acetate for 12 hours. For native tau protein identification, tau was mixed with ddH2O (1:20) and was identified using synapt-G1 mass spectrometer. The instrument was operated in positive ion mode with capillary voltage of 3kV at room temperature.

### HDX microfluidic chip fabrication

A rapid mixing module and proteolysis chamber were integrated on a Poly(methyl methacrylate) (PMMA) chip using VersaLaser as previously described with few modifications [34,35]. Briefly, the proteolytic chamber of dimensions of 28.5mm X 4.9mm X 0.4mm was etched on the chip surface using CO_2_ laser ablation. The rapid mixing and acid quenching capillaries were incorporated in the central channel and adjacent channel respectively (Fig. 1A). Pepsin agarose beads were mounted into the proteolytic chamber and the beads were activated by incubating in 1M HCl for an hour at room temperature. The reactor was then flushed using acetic acid at pH 2.4 to remove the residual 1M HCl. A thin silicon rubber pad was used to create a tight seal between the PMMA chips using custom built clamps. The chip was then interfaced with QSTAR Elite hybrid quadrupole time-of-flight (QTOF) mass spectrometer.

### H/D Exchange of Tau and Phosphotau

100uM tau/phosphotau was mixed with D_2_O (1:3) in the rapid mixing module. This was achieved by adjusting the flowrates of the protein (1μl/min) and D_2_O (3ul/min). To increase the labeling time of deuterium, the position of mixer was adjusted manually. By doing so, labeling times ranging from 42 ms to 12 s were achieved. After the H/D exchange interval, the labeling reaction was quenched by the flow of acetic acid at pH 2.4 from the acid channel. Subsequently, the labeled protein was digested in the proteolytic chamber. Phosphotau was peptically/tryptically digested and phosphopeptides were isolated and enriched using Pierce TiO_2_ phosphopeptide enrichment and cleanup kit. Where possible, the phosphorylation sites were confirmed by MS/MS [32,36].

### Data and Kinetic Analysis

The deuterium uptake percentage by each peptide was calculated using custom built FORTRAN software. Theoretical intrinsic rates that are dependent on primary sequence were calculated using the Sphere web tool [26,29]. As demonstrated in previous studies using the microfluidic device [26,35], back exchange was assumed to be negligible and rapid-exchanging amides (i.e., the N-terminus and side-chains) were assumed to have equilibrated with the electrosprayed solution prior to ionization [29]. The observed and intrinsic deuterium exchange values were plotted as function of labeling time to yield uptake kinetics. Kinetic uptake profiles were then fit to a single exponential function to extract the observed and intrinsic uptake rate constants (*k*_rc_ and *k*_obs_) for each peptide [29]. The ratio *k*_rc_ / *k*_obs_, yields the dimensionless Protection Factor (PF), which represents a semi-quantitative measure of the degree to which a particular segment is structured in the conformational ensemble. It is not clear what effect the presence of phosphate would have on the ‘random coil’ HDX rates at individual sites, thus phosphopeptides with more than a 25% contribution from backbone amide protons adjacent to phosphorylation sites (*i*.*e*., where the presence of phosphate could have a significant impact on the average deuterium uptake rate for the peptide) were discounted in the PF analysis. Only 4% of observed peptides fall into this category.

### Structure modeling

An initial pdb file for the ‘native’ tau structure was selected from the ensemble of structures generated in previous work by the Zweckstetter group [18] based on the Pearson coefficient of a comparison between our HDX data and calculated solvent accessibility. A starting structure for the hyperphosphorylated protein was generated from the selected ‘native’ structure by forced intermolecular repulsion of the backbone. The PDB files were geometrically translated using MMTSB, and hydrogen atoms were added using WHATIF web server (http://swift.cmbi.ru.nl/servers/html/htopo.html). FRODAN v1.0.1 was used to perform geometric molecular simulation in the non-targeted mode, generating 30,000 candidate structures. A PDB snapshot was generated every time the RMSD with the previous model reached 1.0 Å. For each snapshot the accessible surface area (ASA) was calculated using VADAR v1.4. It was run in batch mode and configured to produce ASA for individual atoms. Afterwards, the ASA output was filtered to include only the backbone nitrogen atoms. The N-terminal residue was excluded from the analysis due to its atypically high ASA values. The residues were grouped by the peptides, and the average ASA was computed for each. The quality of the match was evaluated as the Pearson correlation coefficient between the HDX and the mean ASA (only the peptides that included both experimental and theoretical datasets were considered).

## Results

TRESI-HDX was implemented on a custom microfluidic chip that supports a ‘bottom-up’ workflow (Fig. 1A) [26]. In order to examine the low protection factors associated with residual structure in IDPs, the chip was operated at flow-rates resulting in HDX labeling times from 42 ms to 8000 ms, and an average residence time in the digestion microreactor of 6.6 s. Digestion profiles for native and hyperphosphorylated tau were similar, yielding 77.1% and 71.7% sequence coverage, respectively (Fig. 1). The linear shape of these profiles indicates that tau contains no regions that are particularly resistant to proteolysis under acidic conditions (which can arise from the adoption or persistence of structure at low pH) [34]. Peptides and phosphorylation sites were identified by MS/MS where possible, with supporting evidence from known phosphorylation sites. Ultimately, 75 unique peptides were identified from the native protein and 71 from phosphotau containing 19 unique phosphorylation sites (five of which could not be isolated by MS/MS). A full list of the observed peptides and phosphorylation sites is provided in S1 Table.

**Fig 2:**
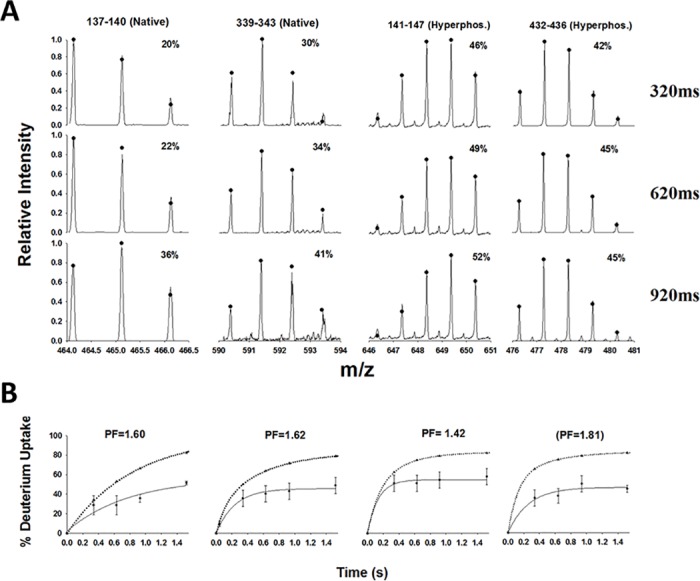
Workflow from raw data to deuterium uptake kinetics for typical peptides from the native and hyperphosphorylated protein. **A**. Raw data for four peptides (columns) each with fits to the isotopic distribution to determine deuterium uptake (filled circles) at three different timepoints (rows). **B**. The resulting kinetic profile for each peptide, with single exponential fit (solid line) to extract *k*_obs_. The calculated ‘random coil’ profile (dotted line, filled triangles) is shown for comparison.

The process of analyzing raw TRESI-HDX data to yield protection factors is summarized in Fig. 2. Deuterium uptake for each peptide at a given labeling time was determined by fitting the observed isotopic distributions to theoretical distributions generated for various uptake levels using a custom FORTRAN program as described previously [37]. The ‘best fit’ distributions are shown in Fig. 2 (filled circles), along with the associated deuterium uptake value. This fitting procedure is required to accurately analyze TRESI-MS data, particularly at early timepoints where low-level labeling often distorts the isotopic distribution without significantly shifting its centroid. Once deuterium uptake has been determined for each peptide at each timepoint, uptake kinetics profiles are generated (Fig. 2B). In ordered proteins, uptake kinetics are often biphasic or ‘stretched’ in part because many peptides originate from regions that include both well-structured and unstructured features [25]. In the current study, we found that all uptake kinetics were well described by a single exponential expression, which is consistent with the expected lack of well-defined structure in tau. Kinetic profiles for all peptides observed at 3 or more labeling times and in all replicates (n = 3) are provided in S1 Fig.

**Fig 3:**
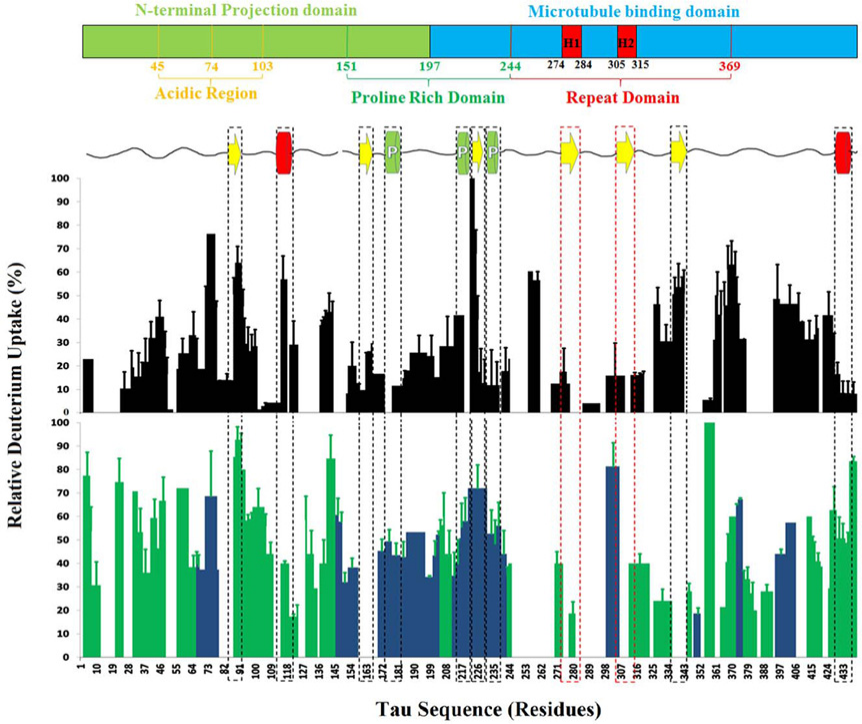
Relative deuterium uptake profiles for native and hyperphosphorylated tau at 1.52 s of D_2_O exposure. The native HDX profile (black bars) is shown directly below the tau domain structure and NMR-derived secondary structure map[18]. On the secondary structure map, yellow arrows indicate β-sheet propensity, green cylinders represent residual polypropylene helices and red cylinders denote regions with significant (> 18%) α-helical propensity. The hexapeptide regions are boxed in red. The hyperphosphorylated HDX profile (green and blue bars) is shown below the native profile. Blue bars indicate the presence of at least one phosphate on the segment indicated.

Single exponential fits to the observed HDX kinetics (Fig. 2B, solid lines) yield the exchange rate constant *k*_obs_ for each peptide. The primary-sequence dependent ‘random coil’ rate constants *k*_rc_ (Fig. 2B, dotted lines), which are needed to calculate segment-averaged PFs as described earlier, are obtained from a multiexponential expression containing the known ‘random coil’ exchange rates for each residue [38]. The N-terminal amino acid is assumed to have undergone rapid back-exchange during proteolysis and is not included in the *k*_rc_ multiexponential, however, residence time in the proteolytic chamber is sufficiently short in our setup that we can incorporate the residue adjacent to the N-terminus (which must also be discounted in most conventional systems) [25,26,38][19]. The uptake profiles shown in Fig. 3 use the direct measurement '% deuterium uptake' at 1.52 s to provide a straightforward picture of relative protection from exchange with maximum sequence coverage.

Native tau exhibited weak, but significant protection throughout the protein, with only 6.7% of the sequence exhibiting negligible protection (PF ≤ 1.2) and 14.6% of the sequence showing moderate protection (PF ≥ 10). No PFs in the range normally associated with stable secondary structure were detected. The protein was 90% exchanged after 10 s of D_2_O exposure, and was fully deuterated in under 2 min. As demonstrated in previous studies involving the TRESI-HDX chip, these measurements are consistent with negligible back-exchange [26,34]. PFs ranged between 1 and 70, with an average PF of 8.1 indicating a weak but widespread network of hydrogen bonds and/or solvent protection consistent with a weakly-ordered globular structure. The deuterium uptake profile for the native protein at 1.52 s is shown in Fig. 3A, with a domain structure and secondary structure map based on a chemical shift index analysis by Mukrasch and co-workers for reference [18]. Significant protection was observed at the N- and C-termini, the central domain and the hexapeptide regions.

Tau was hyperphosphorylated by incubation with GSK-3β/ATP for 36 hours [32] (S2 Fig.), after which TRESI-HDX was conducted immediately. Care was taken to ensure that aggregates were not significantly populated at this time; no soluble aggregates were detected by EM immediately following hyperphosphorylation, and fibrillar aggregates appeared only after an additional 48 hrs of incubation post-hyperphosphorylation (*i*.*e*., 84 hours total incubation, S3 Fig.). Unphosphorylated tau also exhibited fibrillization, but on a substantially longer timescale; sparse fibrillar aggregates with non-PHF morphology were observed after 8 days, suggesting weak nucleation for the native protein [39]. The deuterium uptake profile for GSK-3β hyperphosphorylated tau at 1.52 s is shown in Fig. 3B. The profile exhibits a general increase in deuterium uptake compared to the native protein, particularly at the N- and C-termini and, importantly, in the hexapeptide II region (only a slight increase in uptake occurs at hexapeptide I). There are also two contiguous regions, one bounded by G326 and S352, and the other by L114 and Q124 in which deuterium uptake *decreased* compared to the native protein.

## Discussion

A number of attempts have been made to characterize the native structural ensemble of tau. An overall lack of persistent secondary structure is supported by CD measurements and the radius of gyration (from SAXS) is consistent with little or no tertiary structure apart from ‘folding over’ of the N- and C-termini [40]. Ensemble FRET measurements have suggested a ‘paperclip-like’ global fold [41] while single-molecule experiments suggest an ‘S’ shape [42] (the main difference being the extent to which the termini interact with each other as opposed to the ‘projection’ and ‘repeat’ domains). The most detailed structural data come from NMR, building on an elegant assignment of resonances for the full-length protein (a challenging prospect for any large IDP due to low spectral dispersion) by Mukrasch *et al*. [18]. NMR measurements have allowed predictions of the locations of residual secondary structure [18] and site-specific mapping of ϕ/φ angle ‘conformational potentials’ in a tau fragment [19].

TRESI-HDX data provide the first sub-molecular view of tertiary structure biases in the native full-length tau conformational ensemble. Low deuterium uptake at the termini, the proline rich domain and the hexapeptide region are consistent with the ‘S’-shaped model derived from single-molecule FRET measurements [42], which predicts persistent contact between the N-terminus and the proline rich domain and between the C-terminus and the hexapeptide region. The hexapeptide motifs, corresponding to ^275^VQIINK^280^ (H1) and ^306^VQIVYK^311^ (H2), are the segments of the tau sequence that are most directly linked to amyloidogenic propensity [43]. These motifs have a larger proportion of hydrophobic residues and a substantially higher β-structure propensity than the rest of the tau primary sequence and are thought to form the ‘β-sheet core’ of tau fibrils [44]. Moreover, excision of the hexapeptide motifs virtually eliminates amyloidogenic propensity in tau [43]. Thus, low exchange in the hexapeptide region (indicating sequestration of the hexapeptides) is consistent with the non-amyloidogenic character of native tau *in vivo*.

While the HDX profile agrees with global structural models for native tau, regions predicted to have significant secondary structure *via* the chemical shift index and J-scalar couplings [18], do not generally show a lowered level of exchange. This is a reasonable result for β-stands and polyproline II helices, since these structures are not supported by internal networks of hydrogen bonds, however, of the two regions identified as having residual α-helical structure, only the one at the C-terminus shows a high degree of protection, which is likely a result of tertiary contacts associated with the global fold. Thus, it would appear that in IDPs, factors affecting tertiary structure (*i*.*e*., backbone hydrogen bonding not linked to secondary structure and solvent access) have a greater influence on millisecond HDX than the presence of residual secondary structure. NMR-predicted α-helical regions do exhibit notable behavior in the conformational bias shift that occurs upon hyperphosphorylation, however (see below).

Incubation of tau with GSK-3β/ATP to completion of the phosphorylation reaction generates a heterogeneous mixture of hyperphosphorylated species, with phosphorylation detected at 23.9% of predicted sites for all kinases and 51% for GSK-3β. This is well above the degree of phosphorylation observed in normal mature neurons, where tau typically exhibits < 10% phosphorylation [1,14,45]. All of the unambiguous phosphorylation sites observed match the canonical S/T-XXX-S/T consensus motif for GSK-3β, except for T69 and S305. However, these have been identified previously *in vitro* by DP Hanger *et al*. [46]. Many of the observed phosphorylations are also detected in tau PHF aggregates including S214, S202, S396 and S404 [47,48]. The hyperphosphorylated ensemble was substantially more amyloidogenic than the native protein, exhibiting fibrils consistent with PHF morphology 48hrs after completion of the phosphorylation reaction (S3 Fig.).

While native tau is among the most well-studied IDPs, little is known about the amyloidogenic, hyperphosphorylated protein. Low resolution structural data comparing native and hyperphosphorylated tau suggest that hyperphosphorylation causes the adoption of a more extended structure [49,50][23]. A similar global elongation has also been observed to result from exposure of tau to heparin, which also induces amyloidogenesis [50]. The general increase in deuterium uptake observed in our experiments supports this global picture, however, in the higher-resolution view provided by TRESI-MS, the 'elongation' is clearly associated with release of the native ‘S’ global fold, indicated by a marked increase in deuterium uptake at the N- and C-termini, with similar increases in the proline rich domain and the hexapeptide region (Fig. 3B).

One region that shows a marked *decrease* in deuterium uptake upon hyperphosphorylation is bounded by G326 and S352, corresponding to the microtubule-associated R3 and R4 regions in the repeat domain. This nucleus of new residual structure could be linked to the decreased affinity of hyperphosphorylated tau for microtubules [1]. The other region exhibiting lowered deuterium uptake is ^114^LEDEAAGHVTQ^124^, corresponding exactly to a region identified as having significant (18%) helical structure in the native ensemble [18]. Since adjacent regions exhibit increased deuterium uptake, this decrease may be attributable to heightened -helical prevalence in the hyperphosphorylated ensemble. A similar effect is observed at the C-terminus, where the presence of residual helical structure appears to mute the increase in deuterium uptake associated with release of the global fold. Of the two hexapeptides, H2 shows by far the greatest increase in deuterium uptake, going from strongly protected in the native ensemble to completely exposed in the hyperphosphorylated ensemble. This points to a dominant role for H2 in GSK-3β mediated increases in tau amyloidogenic propensity.

**Fig 4:**
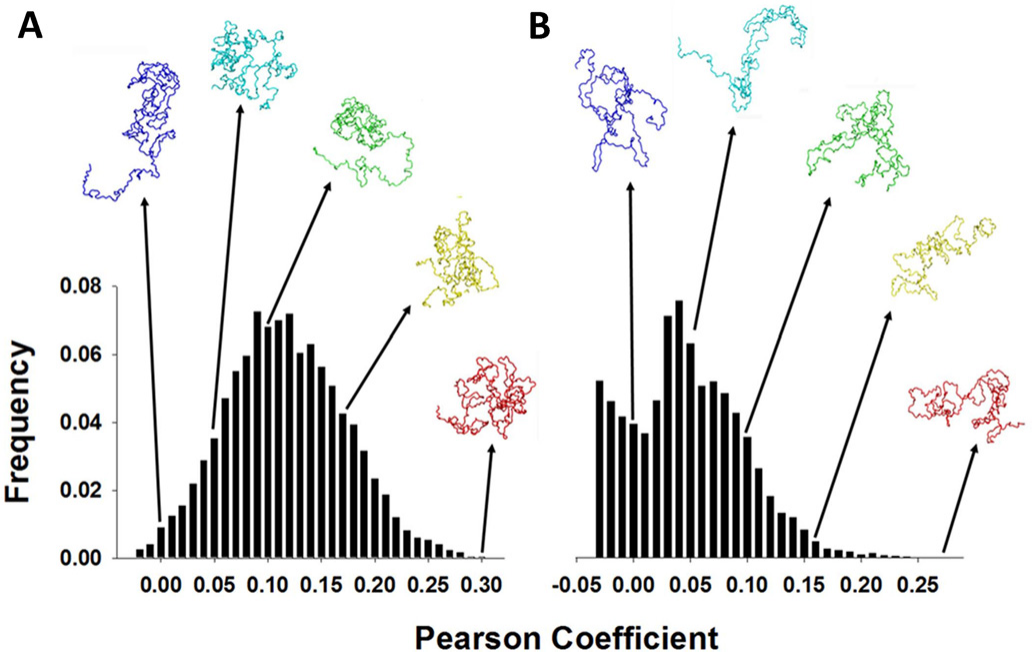
Histograms showing the distribution of agreement with the HDX data (Pearson coefficient) within the FRODAN ensembles. Each ensemble consists of 30,000 candidate structures, initialized based on pdb coordinates provided by the Zweckstetter group [18], and optimized using the FRODAN algorithm. Examples of structures associated with various R-value ‘bins’ are shown above, with the ‘most representative’ structures at the far right. **A**. The native ensemble. **B**. The hyperphosphorylated ensemble.

In order to characterize the structural ensembles that are predicted from our HDX profiles, we have generated theoretical ensembles for native and hyperphosphorylated tau using Framework Rigidity Optimized Dynamics Algorithm New (FRODAN), a low computational complexity alternative to MD [51]. This approach allows us to build ensembles that span an exceptionally broad region of conformational space, which is critical to accurately represent IDPs. The extent to which individual structures within the FRODAN ensemble reflected the actual native and hyphosphorylated tau ensembles was determined by matching Volume-Area-Dihedral Angle Reporter (VADAR)-calculated, backbone amide-specific solvent accessibility [52] of the model with '% uptake' in our HDX profiles (Fig. 4). The comparison is expressed ultimately as the Pearson correlation coefficient *R* between an individual FRODAN model’s solvent accessibility profile and the experimentally-derived HDX profile. A similar approach has been shown to be highly predictive of HDX in weakly structured regions of proteins [53].

**Fig 5:**
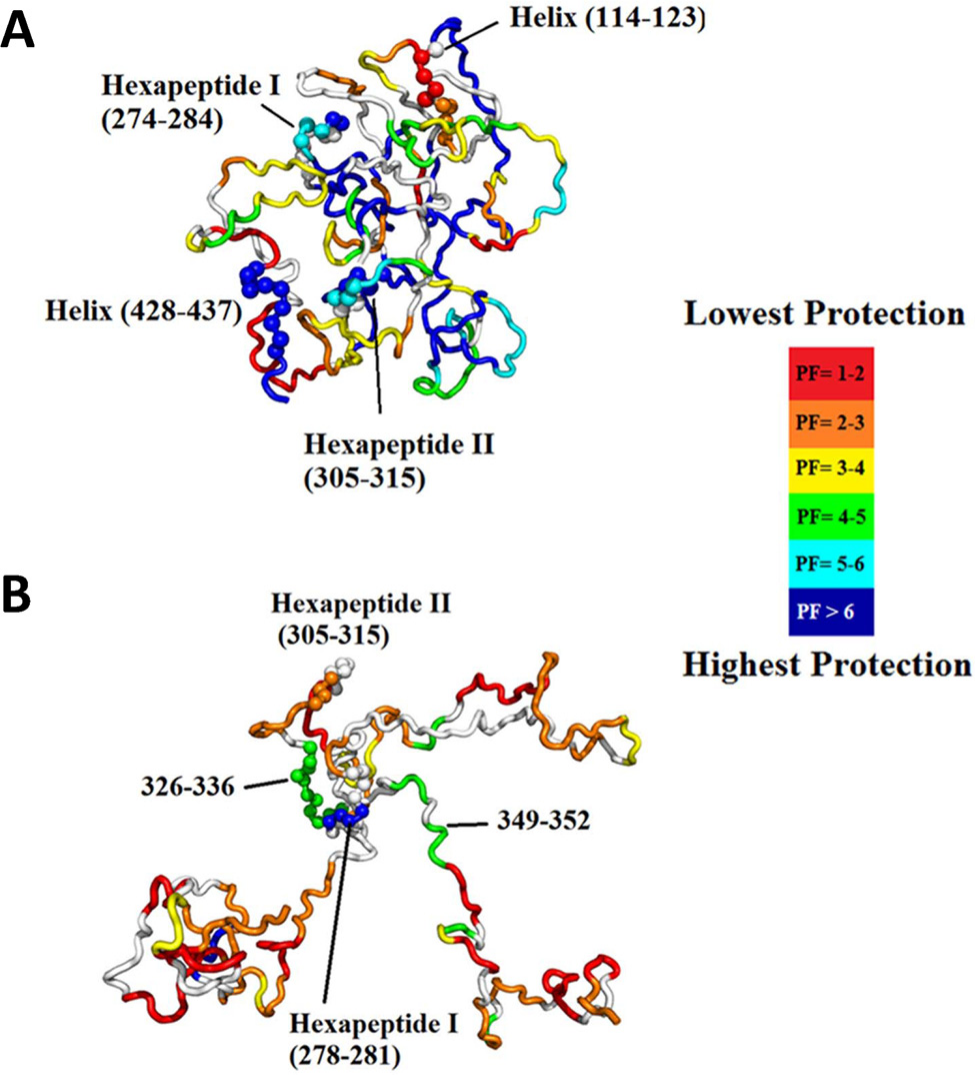
Most representative structures from the native and hyperphosphorylated ensembles, colored by PF. **A**. Most representative structure for the native ensemble (*R* = 0.30), which exhibits a global ‘S’-shaped fold with sequestration of the hexapeptides. **B**. Most representative structure for the hyperphosphosphorylated ensemble (*R* = 0.29) showing release of the N- and C-termini, full exposure of H2 and a few regions of residual structure, including around H1.

As would be expected for an IDP, no single model is exceptionally well correlated to the ensemble TRESI-HDX data (*R*_max_ = 0.3). Nonetheless, the ‘most representative’ structures exhibit all of the features predicted from a qualitative evaluation of the TRESI-HDX profiles. High-ranking structures for the native protein (corresponding to *R* > 0.275 or the top 10%) are universally compact with many exhibiting precisely the expected global ‘S’-fold (Fig. 4A) and all showing complete sequestration of the hexapeptides. High-ranking structures for the hyperphosphorylated protein (corresponding to *R* > 0.16 or the top 10%) are all extended with a few remaining structural nodes surrounding regions of relatively low deuterium uptake (Fig. 4B). Many of these top-ranked hyperphosphorylated structures clearly show full exposure of H2 with sequestration of H1. In Fig. 5, deuterium uptake at 1.52 s is mapped on to the highest-scoring structures from the native and hyperphosphorylated FRODAN ensembles. While it is clear that these individual models do not perfectly reflect the ensemble, they nonetheless provide an accurate representation of the conformational bias shift that underlies increased amyloidogenic propensity in GSK-3β hyperphosphorylated tau.

In summary, we have obtained an unprecedentedly detailed view of GSK-3β hyperphosphorylation-driven conformational shifts in the full length (441 residue) IDP tau. Many of the global and local structural changes are easily rationalized as increasing amyloidogenic propensity in the GSK-3β hyperphosphorylated tau monomer. Specifically, hyperphosphorylation results in release of the global fold, development of new intramolecular interactions in the microtubule binding region and greatly increased exposure of H2, however, H1, which has also been implicated in amyloidogenesis, remains largely sequestered. These data provide structural evidence for a causative role for hyperphosphorylation in tau amyloidogenesis *in vivo* and illustrate the power of TRESI-HDX as an analytical tool for characterizing pathogenic conformational shifts in IDPs.

## Supporting Information

S1 Table All peptides and phosphopeptides detected in three or more replicates from native and hyperphosphorylated tau, with associated protection factors. (DOCX)

S1 Fig Kinetic deuterium uptake profiles for all peptides from the native and hyperphosphorylated protein that were observed in at least 3 time points and all replicates (n = 3). Intrinsic rates are shown as dashed lines and single exponential fits to the experimental data are shown as a solid line. Residue numbers and protection factor of are provided at the top of each plot.
(DOCX)

S2 Fig Gels showing the shift to high apparent mass associated with phosphorylation of tau after 5 hours and 30 hours. No additional phosphorylation was observed beyond 30 hours.
(DOCX)

S3 Fig Typical SEM image of an early insoluble aggregate of tau at 56 hours after completion of phosphorylation. Smaller paired helical filaments were observed as early as 48 hours after completion of phosphorylation.
(DOCX)

S4 Fig MS/MS spectra for selected phosphopeptides. (DOCX)
