# Supplementary material for: Hyperphosphorylation of Intrinsically Disordered Tau Protein Induces an Amyloidogenic Shift in Its Conformational Ensemble
Source: PLoS One. 2015 Mar 13;10(3):e0120416. doi: 10.1371/journal.pone.0120416 (PMC4359001; doi:10.1371/journal.pone.0120416)
Supplement: S1 Table — (DOCX) [file pone.0120416.s001.docx]

**Supplemental Table S1**

|  | **Native** | | **Hyperphosphorylated*** | |
| --- | --- | --- | --- | --- |
| **#** | **Peptide** | **PF** | **Peptide** | **PF** |
| **1** | (A)/EPRQEF/(E)3-8 | 6.4±2.9 | (A)/EPRQE/(F) 3-7 | 1.2±0.3 |
| **2** | (R)KDQGGYTMHQDQ/(E) 24-35 | 4.9±1.2 | (Q)/EFEVME/(D) 7-12 | 2.6±0.6 |
| **3** | (R)KDQGGY/(T) 24-29 | 70.0±7 | (L)/GDRKD(Q) 21-25 | 1.3±0.12 |
| **4** | (M)HQDQEGDT(D) 32-39 | 3.6±0.9 | (T)MHQDQE/(G) 31-36 | 1.4±0.13 |
| **5** | (E)/GDTDAGLKESPLQT(P) 37-50 | 2.7±0.5 | (Q)/DQEGDTDAGLKE/(S) 34-45 | 2.7±0.6 |
| **6** | (D)AGLKESP(L) 41-47 | 1.7±0.2 | (D)AGLK(E) 41-44 | 1.6±0.15 |
| **7** | (L)/QTPTE/(D)49-53 | 69.0±7 | (E)/SPLQ/(T) 46-49 | 1.2±0.2 |
| **8** | (E)/AAALIGDEPLENYLDTE/(Y) 56-72 | 5.4±2.3 | (G)SEEPGSE/(T) 56-62 | 1.4±0.07 |
| **9** | (S)EEPGSETSDAK(S)57-67 | 3.0±0.8 | (E)/TSDAKS(T) 63-68 | 3.2±0.4 |
| **10** | (E)/TSDA/(K) 63-66 | 1.9±0.3 | (A)/KSTpPTAEDVTAPL/(V) 67-79 PHOS | 2.7±0.05 |
| **11** | (T)AEDVTAP(L) 72-78 | 1.3±0.13 | (P)TAED(V) 71-74 | 1.0+0.02 |
| **12** | (A)/PLVDEGAPGKQAAAQPHTE/(I)78-96 | 7.0±2.33 | (T)AEDVTAP(L) 72-78 | 1.0±0.07 |
| **13** | (L)/VDEGAP(G)80-85 | 10.0±1.6 | (Q)/AAAQ/(P) 89-92 | 1.1±0.05 |
| **14** | (V)DEGAPG(K) 81-86 | 7.4±0.25 | (A)/QPHTEIPEGTTAEEA/(G) 92-106 | 1.1±0.05 |
| **15** | (K)QAAAQPHTEIPEGT(T)88-101 | 1.5±0.2 | (H)TEIP(E) 95-98 | 1.1±0.1 |
| **16** | (K)QAAAQP(H) 88-93 | 1.2±0.16 | (E)/IPEGTTAEE/(A) 97-105 | 1.6±0.5 |
| **17** | (Q)/AAAQ/(P) 89-92 | 1.0±0.1 | (G)TTAEEA/(G) 101-106 | 4.2±1.4 |
| **18** | (Q)/PHTEIP(E)93-98 | 3.2±0.21 | (E)/AGIGD(T) 106-110 | 2.1±0.2 |
| **19** | (H)TEIP(E) 95-98 | 3.0±0.47 | (E)/DEAA/(G) 116-119 | 2.5±0.01 |
| **20** | (E)/IPEGTTAEEA/(G)97-106 | 66.1±6.6 | (A)/GHVTQ/(A) 120-124 | 7.9±0.6 |
| **21** | (A)/EEAGIGDTPSL/(E) 104-114 | 23.3±5.6 | (V)SKSKD(G) 129-133 | 2.3±0.8 |
| **22** | (L)/EDEA/(A)115-118 | 1.3±0.18 | (S)KSKDGT(G) 130-135 | 3.4±0.37 |
| **23** | (A)/GHVTQ/(A) 120-124 | 2.5±0.6 | (G)SDDK(K) 137-140 | 2.3±0.5 |
| **24** | (G)SDDK(K)137-140 | 1.6±0.04 | (K)KAKGADG(K) 141-147 | 1.4±0.15 |
| **25** | (D)DKKAKG(A)139-144 | 3.3+0.9 | (K)KAKGADGKT(K) 141-149 | 1.0±0.12 |
| **26** | (D)KKAK(G)140-143 | 1.9±0.26 | (A)/DGKT(K) 146-149 | 2.4±0.18 |
| **27** | (I)ATPRGAAP(P) 152-159 | 11.7±0.8 | (A)/DGKTpKIATPRGAA/(P) 146-158 PHOS | 3.0±0.3 |
| **28** | (A)/TPRGA/(A) 153-157 | 2.3±0.45 | (A)/TpPRGAA/(P) 153-158 PHOS | 2.4±0.18 |
| **29** | (A)/APPGQK(G)158-163 | 2.8±0.5 | (T)RIPAKTp?PPAPKTp?P(P) 170-182 PHOS | 2.2±0.25 |
| **30** | (A)/APPGQKGQ/(A)158-165 | 7.8±0.3 | (A)/KTp?PPAPKTp?PPSSpGEPPKSGD RSp?GYSp?SpPGSp?(P) 174-202 (PHOS 4X) | 1.9±0.25 |
| **31** | (P)PGQKGQA/(N) 160-166 | 10.1+1 | (P)APKTPPSS(G) 178-185 | 3.1±0.16 |
| **32** | /KGQA/(N) 163-166 | 1.1±0.1 | (G)YSSP(G) 197-200 | 4.1±0.1 |
| **33** | (Q)/KGQANATRIPA/(K)163-173 | 8.8±1.8 | (P)GSpPG(T) 201-204 PHOS | 3.0±0.23 |
| **34** | (P)APKTPPSS(G)178-185 | 8.8±0.42 | (S)PGTP(G) 203-206 | 2.4±0.07 |
| **35** | (P)SSGEPPKSGDRSGY/(S) 184-197 | 5.4±1.5 | (S)PGTPG(S) 203-207 | 1.3±0.16 |
| **36** | (E)/PPKSGDRSGYSSPG(S) 188-201 | 4.4±0.9 | (T)PGSRSRTPSL/(P) 206-215 | 1.7±0.29 |
| **37** | (Y)/SSPGSPGTPGSRSRT(P)198-212 | 6.4±2 | (R)TPSpL/(P) 212-215 PHOS | 4.0±0.04 |
| **38** | (G)TPGSRSRTPSLPTP(P)205-218 | 3.8±0.8 | (P)SpLPT(P) 214-217 PHOS | 3.6±0.8 |
| **39** | (R)EPKKV(A)222-226 | 2.1+0.5 | (P)TPPT(R) 217-220 | 2.1±0.4 |
| **40** | (K)KVAV(V) 225-228 | 2.0±0.4 | (P)TpPPTp?REPKKVAVVRTpPPKSp?P Sp?SAKSp?R(L) 217-242 (PHOS 3X) | 1.3±0.16 |
| **41** | (K)VAVVRTPPKSPSS(A)226-238 | 3.7±1.2 | (T)PPKSP(S) 232-236 | 3.0±0.15 |
| **42** | (V)AVVRTPP(K) 227-233 | 2.3±0.02 | (S)PSSAKSRLQTA/(P) 236-246 | 3.0±0.13 |
| **43** | (V)AVVRT(P) 227-231 | 7.0+0.03 | (A)/KSRL/(Q) 240-243 | 2.7±0.1 |
| **44** | (A)/KSRL/(Q) 240-243 | 4.9±1 | (Q)/PGGGK(V) 270-274 | 2.5±0.3 |
| **45** | (A)/KSRLQ/(T)240-244 | 4.4±0.02 | (I)INKK(L) 278-281 | 6.3±1.5 |
| **46** | (K)NVKSKIG(S) 255-261 | 2.4±0.1 | (K)HVPGGGS(V) 299-305 | 1.3±0.18 |
| **47** | (K)SKIG(S)258-261 | 1.8±0.7 | (K)HVPGGGSpV(Q) 299-306 PHOS | 1.0±0.07 |
| **48** | (K)HQPGGGKVQII(N)268-278 | 7.8±3 | (K)PVDLSKVTSKCG(S) 312-323 | 2.3±0.19 |
| **49** | (G)GKVQ/(I) 273-276 | 3.4±0.38 | (L)/GNIHHKPGGGQ/(V) 326-336 | 4.2±0.75 |
| **50** | (S)NVQSKCGSKD(N) 286-295 | 35.2±3.5 | (L)/DFK(D) 345-347 | 3.6±1.6 |
| **51** | (K)HVPGGGSVQIV(Y)299-309 | 6.5±0.8 | (D)RVQSp(K) 349-352 PHOS | 11.0±2.3 |
| **52** | (P)VDLSKVTS(K)313-320 | 4.9±0.03 | (I)GSLDNI(T) 355-360 | 1.1±0.04 |
| **53** | (P)VDLSK(V) 313-317 | 5.8±0.03 | (V)PGGGN(K) 364-368 | 4.7±1.8 |
| **54** | (P)VDLSKV(T)313-318 | 5.0+0.2 | (E)/TpHKL/(T) 373-376 PHOS | 1.4±0.02 |
| **55** | (L)/GNIHHKPGGGQVEVKSE/(K)326-342 | 2.0+0.24 | (L)/TFRE/(N) 377-380 | 3.0±0.37 |
| **56** | (Q)/VEVKS(E)337-341 | 1.1+0.1 | (T)FRENA/(K) 378-382 | 2.9±0.45 |
| **57** | (E)/VKSEK(L) 339-343 | 1.6+0.2 | (E)/NAKA/(K) 381-384 | 5.0±0.25 |
| **58** | (K)IGSLDN(I) 354-359 | 17.5±0.2 | (T)DHGAEIV(Y) 387-393 | 2.8±0.3 |
| **59** | (S)LDNITHV(P)357-363 | 23.3±0.23 | (Y)/KSp?PVVSp?(G) 395-400 PHOS | 1.4±0.09 |
| **60** | (N)ITH(V)360-362 | 1.7±0.3 | (S)GDTSpPR(H) 401-406 PHOS | 1.7±0.28 |
| **61** | (I)THVP(G) 361-364 | 1.7±0.3 | (S)STGSI(D) 413-417 | 1.7±0.22 |
| **62** | (G)GGNKKIETH(K)366-374 | 1.8±0.3 | (G)SIDMV(D) 416-420 | 2.0±0.12 |
| **63** | (G)NKKI(E) 368-371 | 1.1±0.1 | (S)IDMVD(S) 417-421 | 2.6±0.04 |
| **64** | (N)KKIET(H) 369-373 | 2.0±0.4 | (Q)/LATLADEVSASL/(A) 425-436 | 3.4±0.28 |
| **65** | (E)/THKLTF/(R) 373-378 | 1.9±0.3 | (L)/ATLA/(D) 426-429 | 1.0±0.02 |
| **66** | (V)YKSP(V)394-397 | 1.5±0.2 | (L)/ATLADEVSA/(S) 426-434 | 1.5±0.23 |
| **67** | (P)VVSGDTSPRHLSNV(S) 398-411 | 2.3±0.25 | (L)/ADEVSASLAKQGL 429-441 | 1.8±0.06 |
| **68** | (P)VVSGDTSPRHLSNVSSTGSI D(M)398-418 | 4.6±2 | (A)/DEVSA/(S) 430-434 | 1.6±0.13 |
| **69** | (H)LSNVSSTGSIDM(V) 408-419 | 2.8±0.17 | (E)/VSASL/(A) 432-436 | 1.8±0.12 |
| **70** | (L)/SNVSSTGS(I) 409-416 | 5.4±0.32 | (E)/VSASLA/(K) 432-437 | 4.7±1.4 |
| **71** | (D)SPQLATLADE/(V) 422-431 | 1.5±0.3 | (L)/AKQGL 437-441 | 2.5±0.03 |
| **72** | (T)LADEVSASLAKQGL 428-441 | 17.5±0.9 |  |  |
| **73** | (L)/ADEVSASLAKQGL 429-441 | 9.4±0.5 |  |  |
| **74** | (E)/VSASLA/(K) 432-437 | 10.1±1 |  |  |
| **75** | (A)/SLAKQGL 435-441 | 11.7±2 |  |  |

* Unambiguous phosphorylation sites are labeled as (X)p where X corresponds to the phosphorylated residue. Residues marked (X)p? represent possible phosphorylation sites that could not be definitively identified by MS/MS. Where multiple phosphorylation sites are present, (PHOS *n*X) indicates the number of phosphorylations observed on the peptide where *n* is the number of phosphoryl groups.
