# Supplementary figures and images for: Hyperphosphorylation of Intrinsically Disordered Tau Protein Induces an Amyloidogenic Shift in Its Conformational Ensemble
Source: PLoS One. 2015 Mar 13;10(3):e0120416. doi: 10.1371/journal.pone.0120416 (PMC4359001; doi:10.1371/journal.pone.0120416)

**
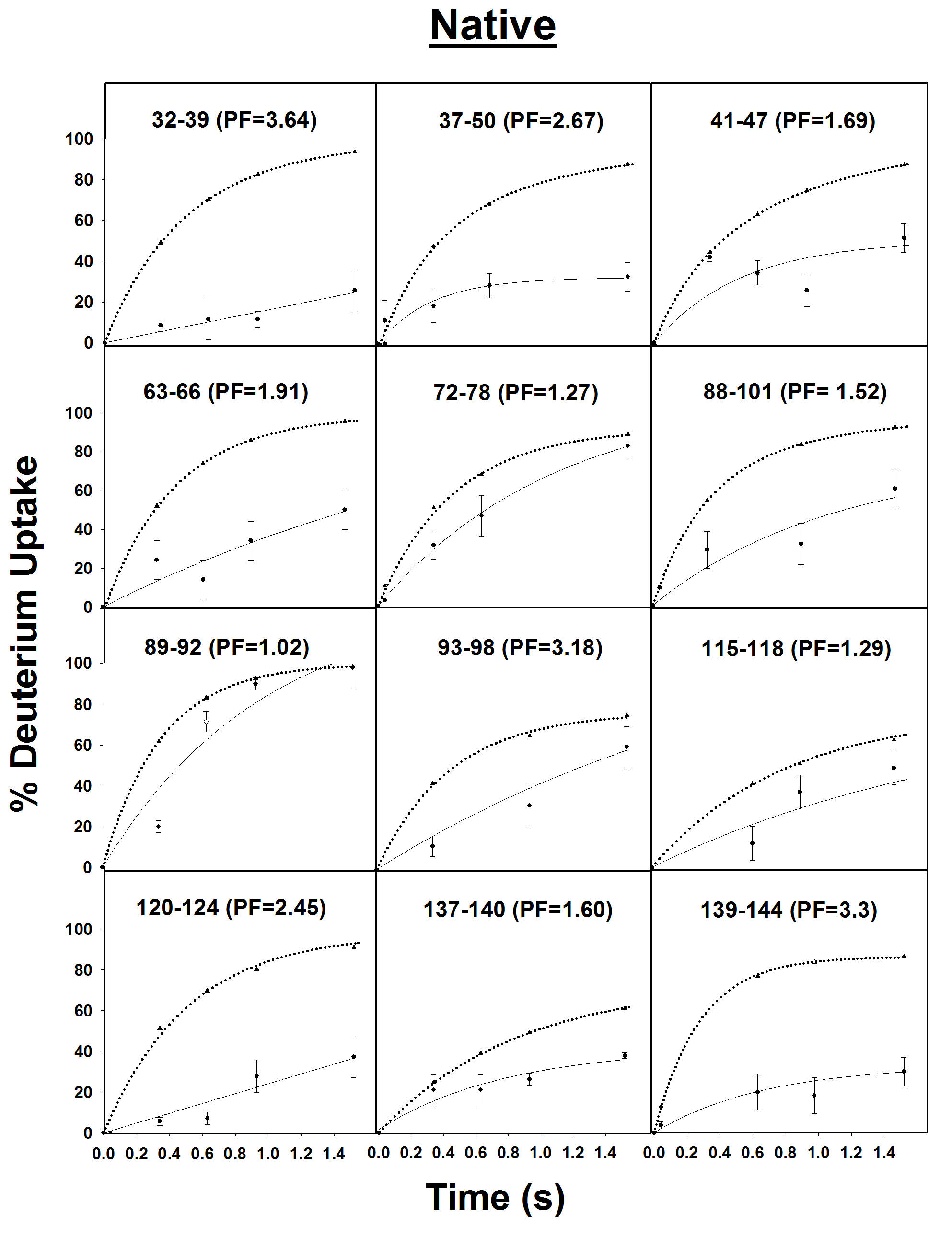
Supplemental Figure S1**


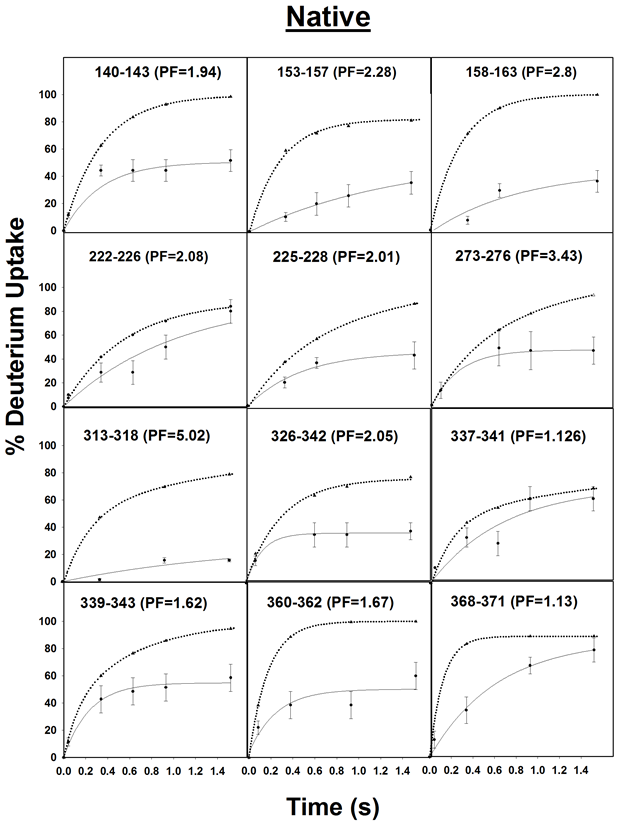


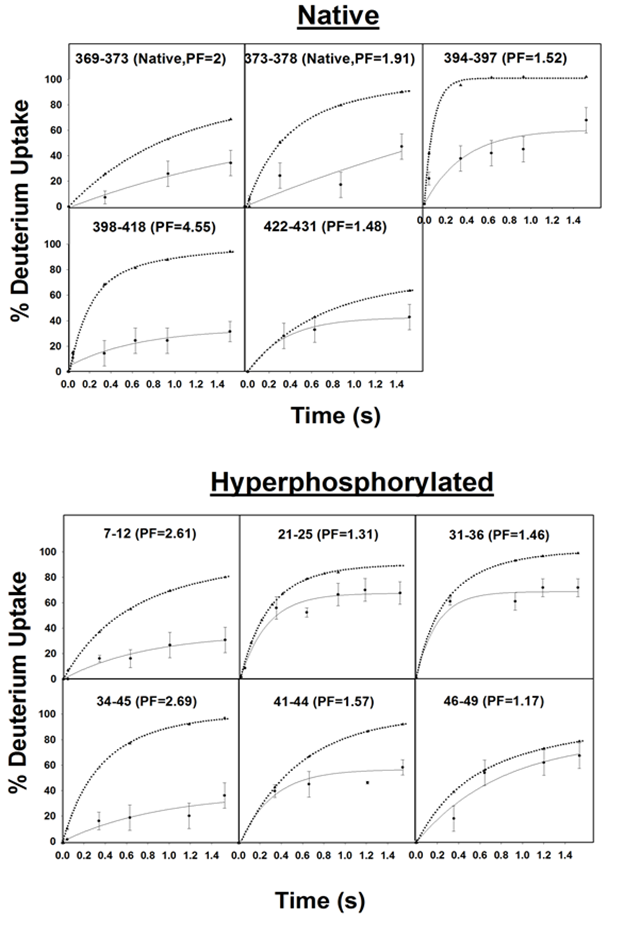


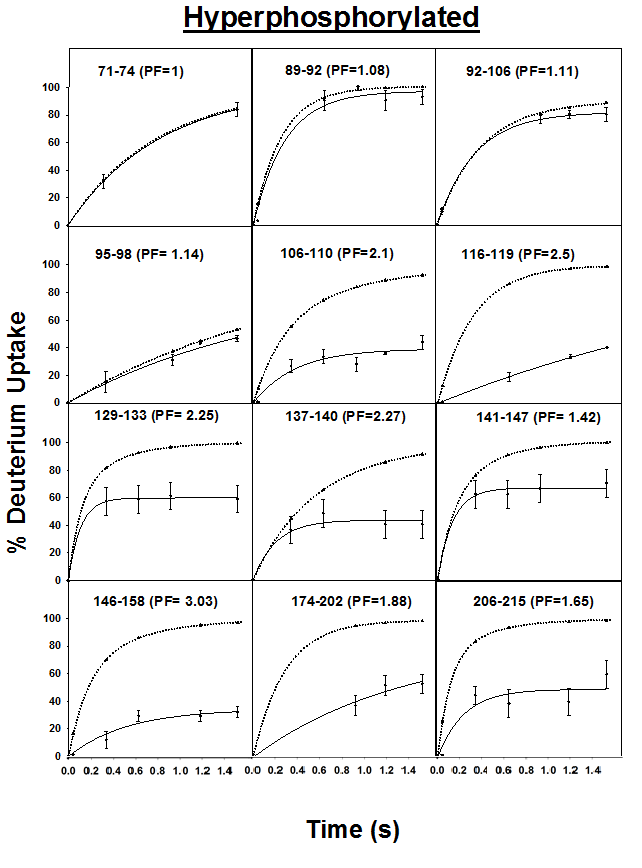


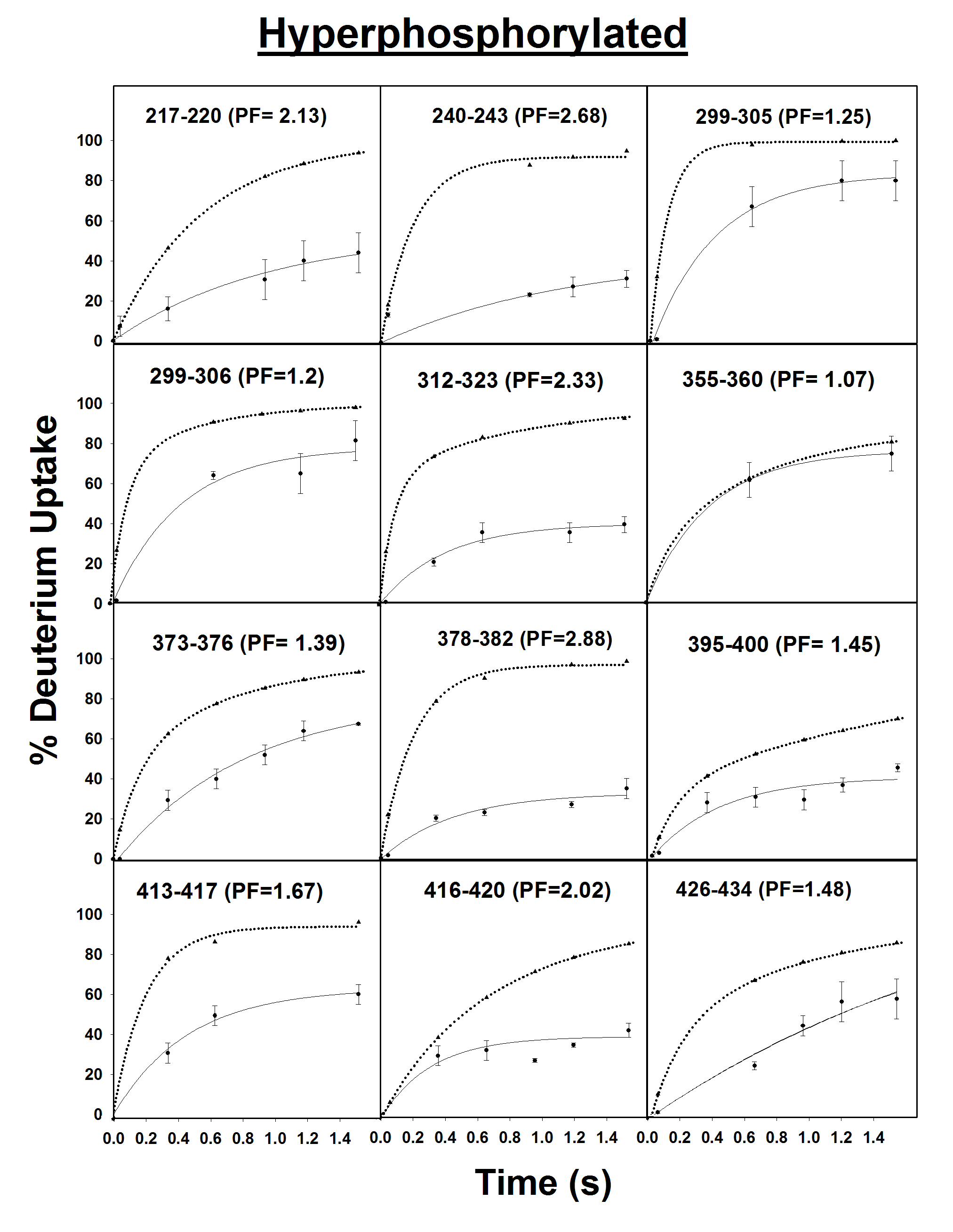


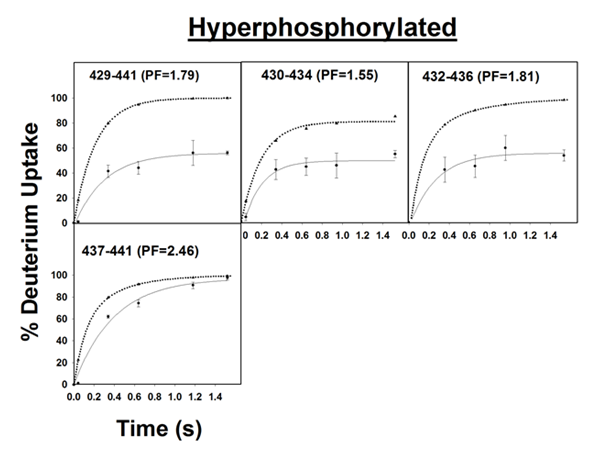

Supplement: S1 Fig — Intrinsic rates are shown as dashed lines and single exponential fits to the experimental data are shown as a solid line. Residue numbers and protection factor of are provided at the top of each plot. (DOCX) [file pone.0120416.s002.docx]

**Supplemental Figure S2**

**
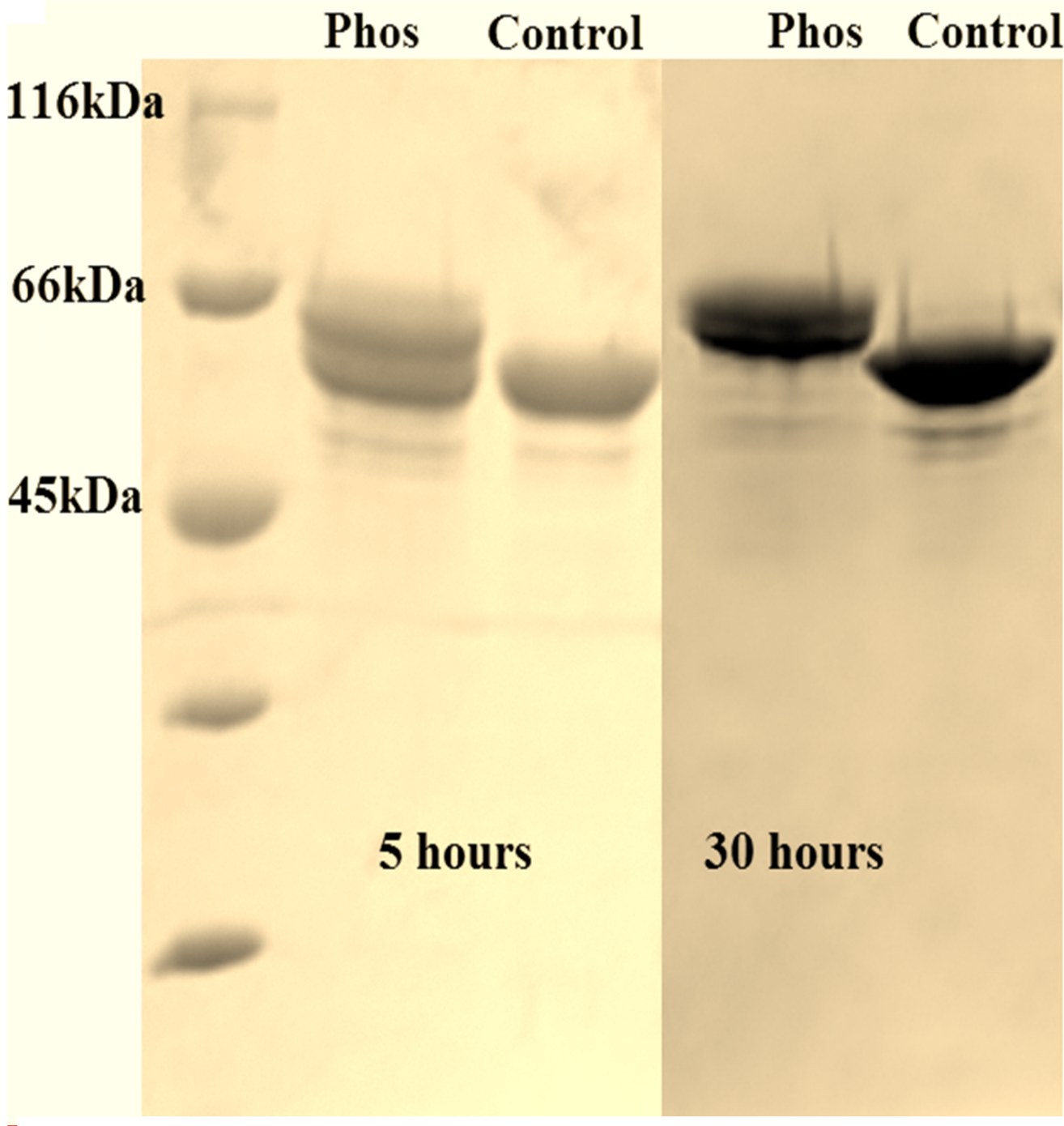
**

Supplement: S2 Fig — No additional phosphorylation was observed beyond 30 hours. (DOCX) [file pone.0120416.s003.docx]

**Supplemental Figure S3**


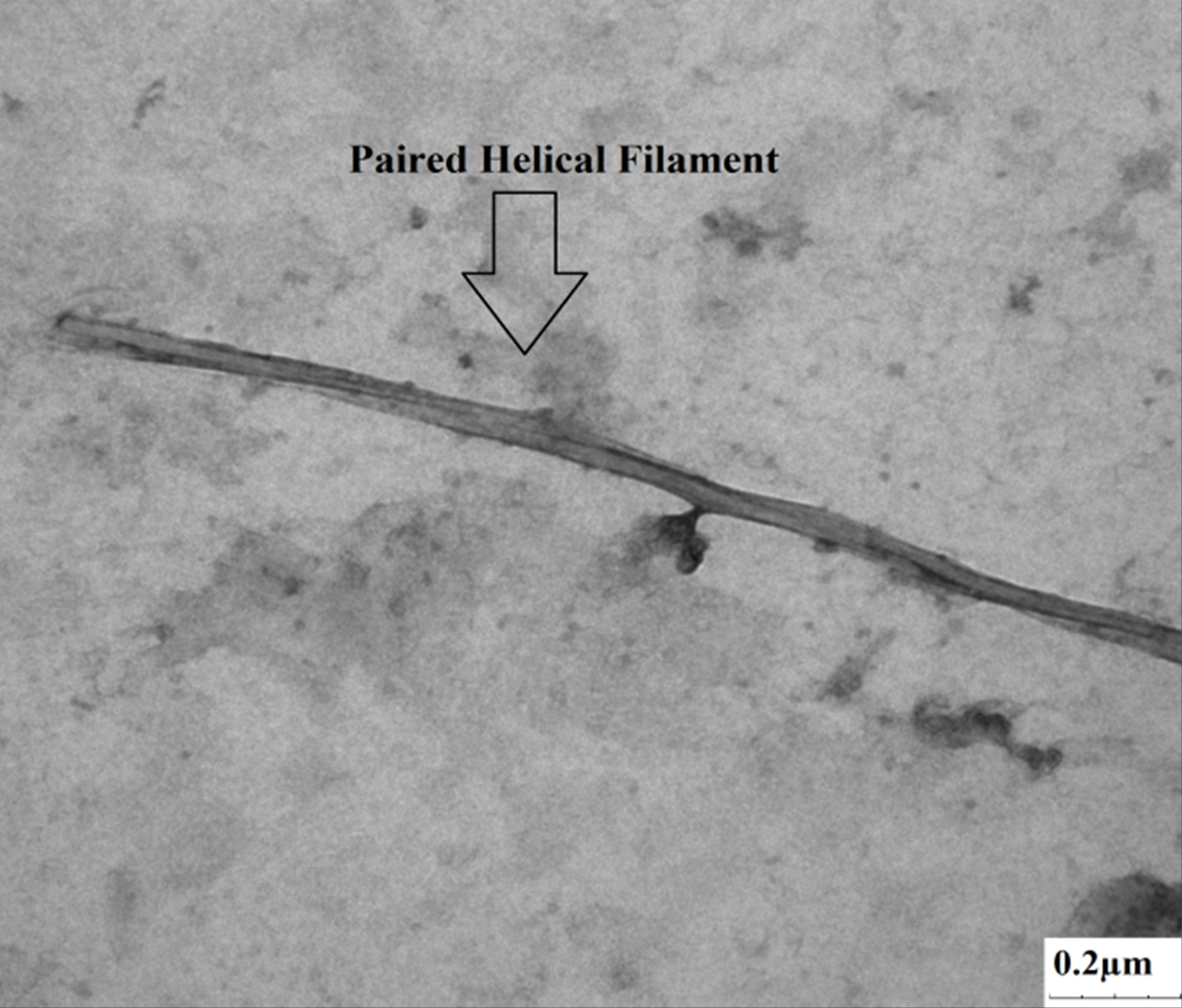

Supplement: S3 Fig — Smaller paired helical filaments were observed as early as 48 hours after completion of phosphorylation. (DOCX) [file pone.0120416.s004.docx]

**Supplemental Figure S4**


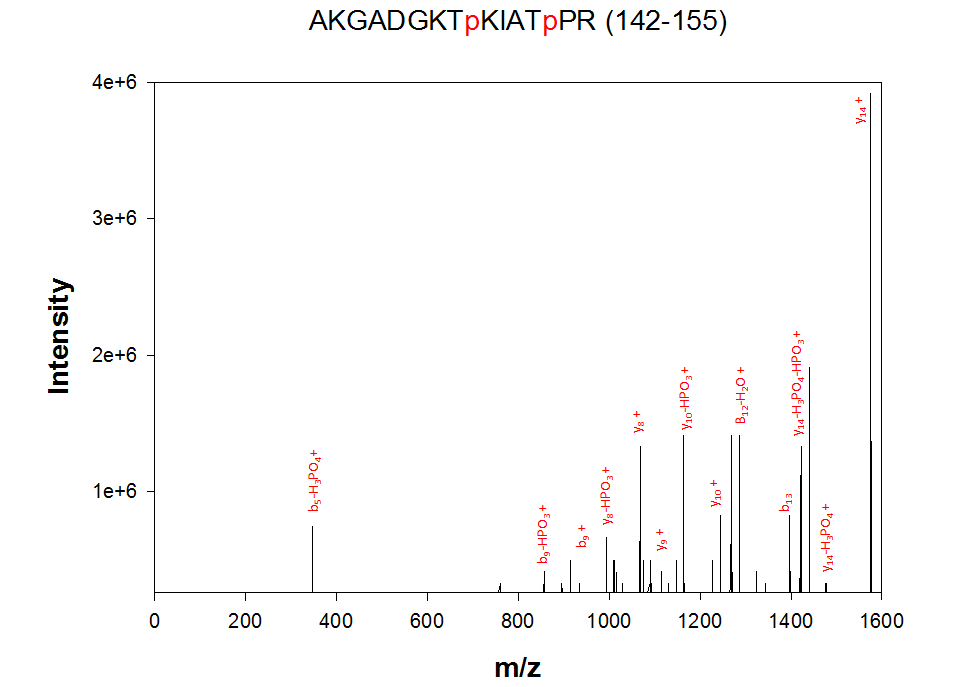


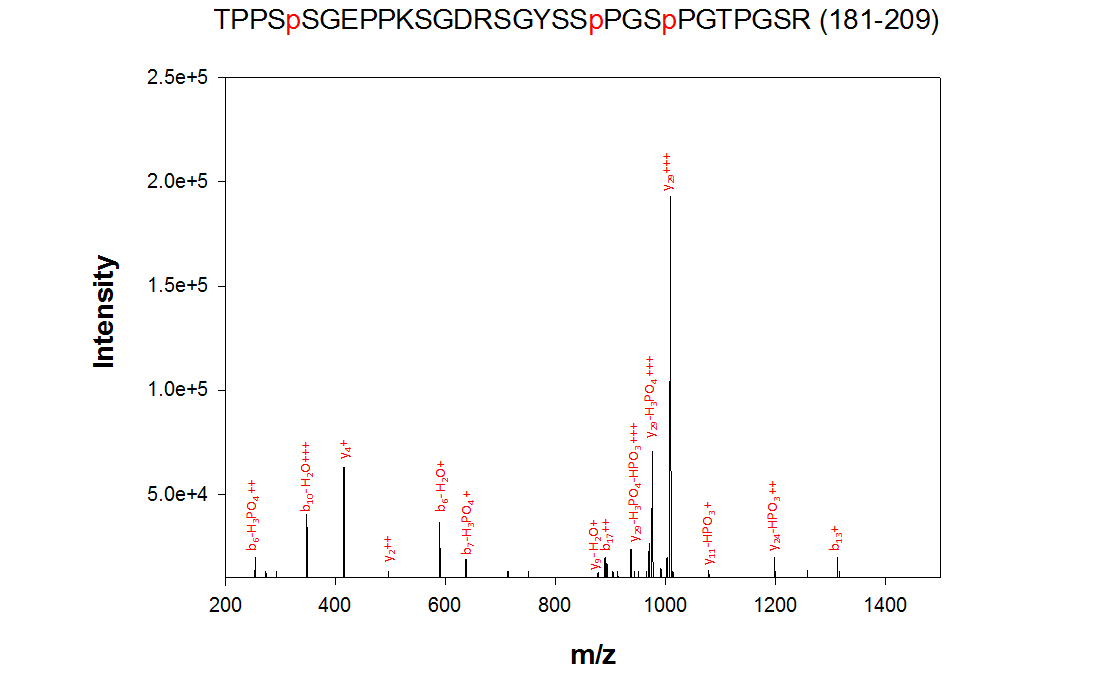


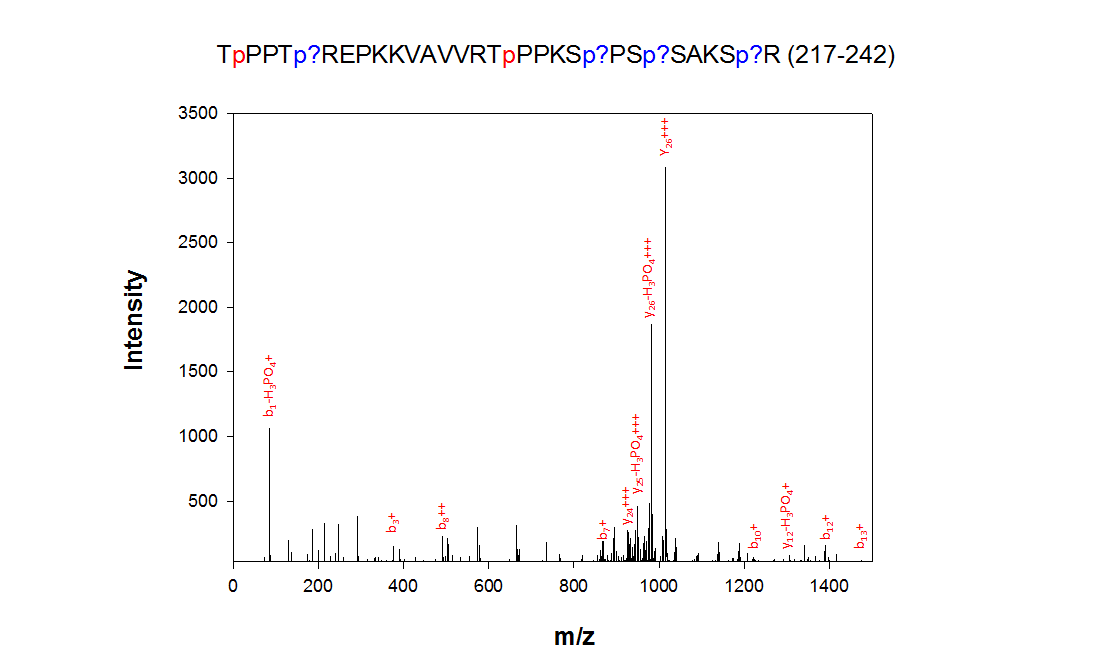


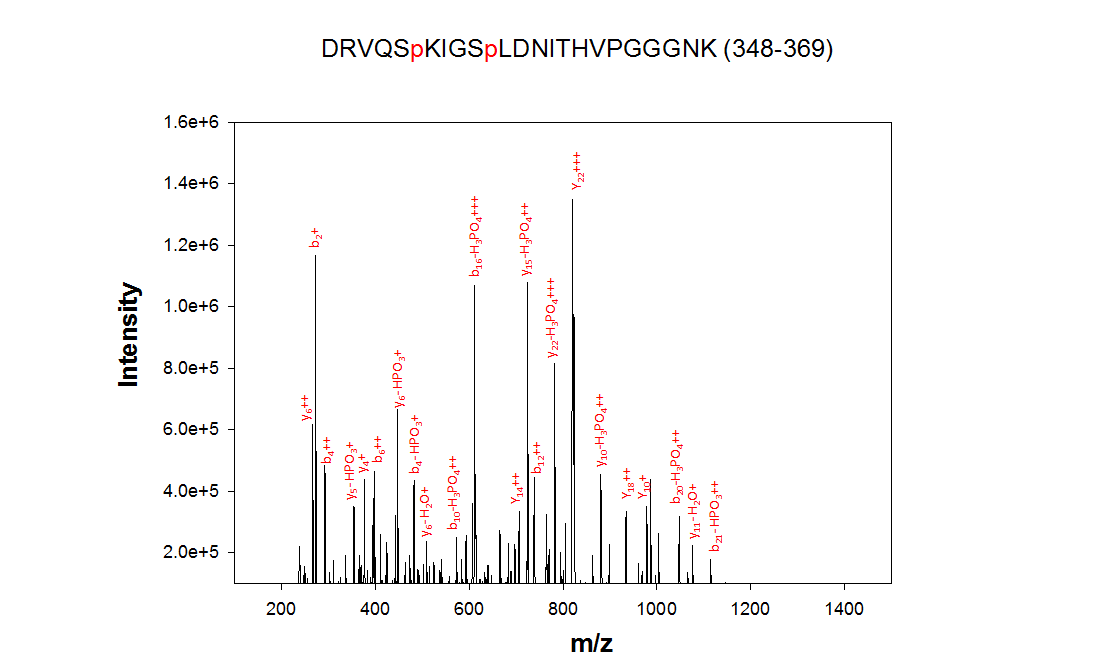


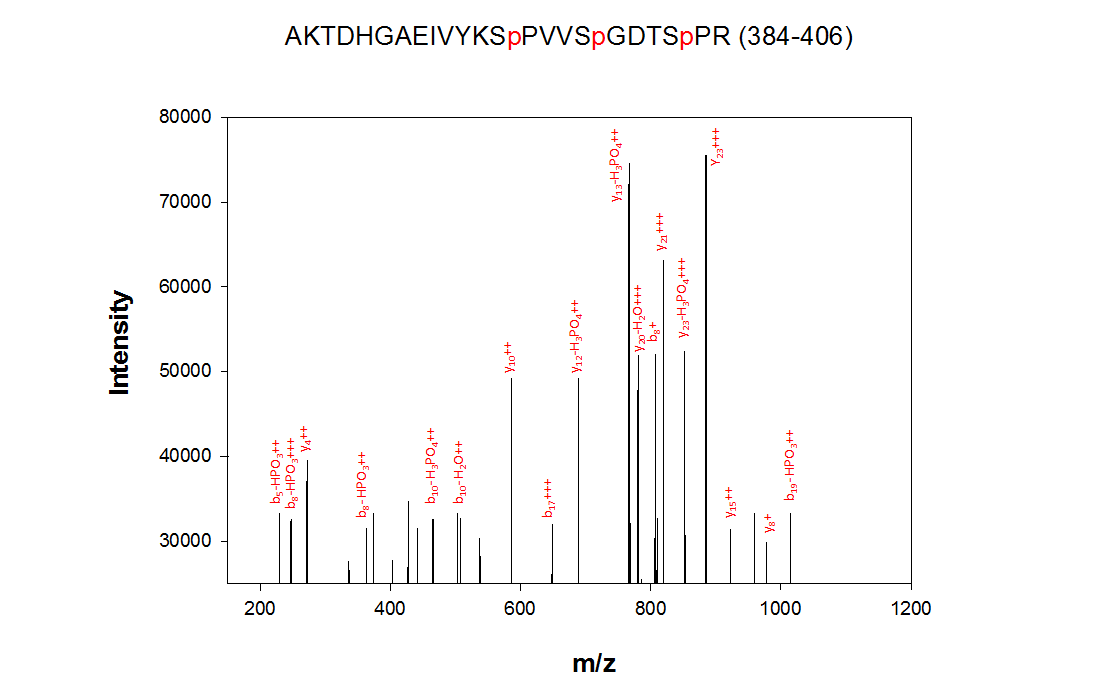


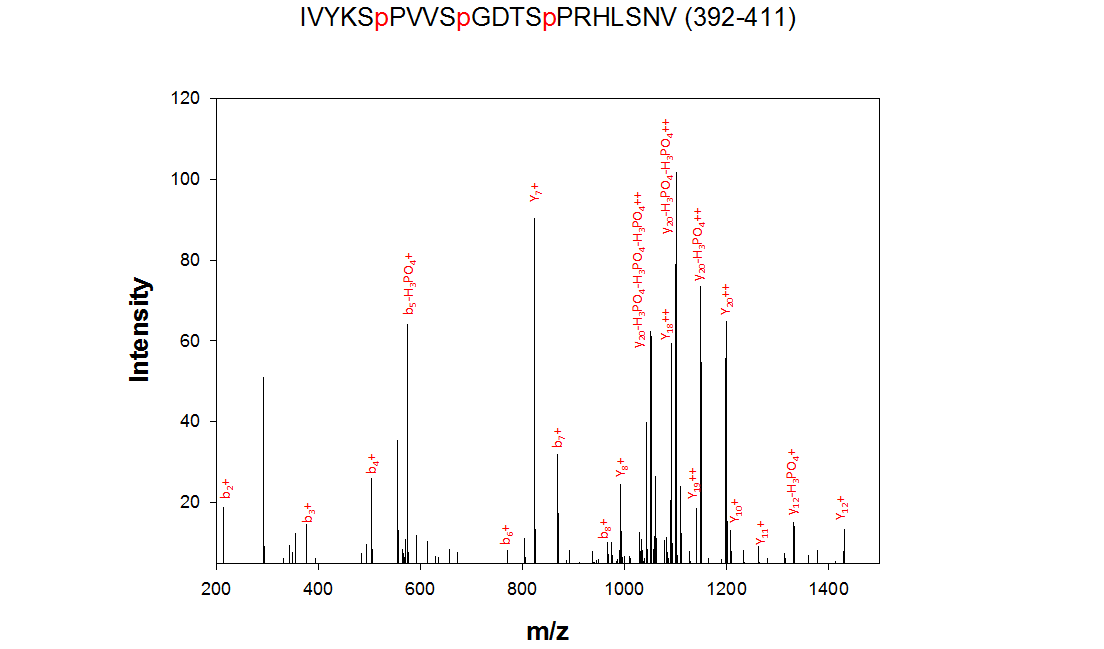

Supplement: S4 Fig — (DOCX) [file pone.0120416.s005.docx]
